# Progress of Ionogels in Flexible Pressure Sensors: A Mini-Review

**DOI:** 10.3390/polym17081093

**Published:** 2025-04-18

**Authors:** Huaning Jiang, Yuqiang Cheng, Xingying Zhang, Mengqing Li, Qinqin Wang, Liang Yang, Changgeng Shuai

**Affiliations:** 1 Institute of Noise and Vibration, Naval University of Engineering, Wuhan 430030, China; d24182403@nue.edu.cn (H.J.);; 2 No. 32281 Unit of PLA, Chengdu 610200, China; 3 No. 91697 Unit of PLA, Qingdao 266000, China; yangliang0306@163.com

**Keywords:** ionogel, flexible sensor, pressure sensor, research progress, wearable electronic device, intelligent robot, healthcare

## Abstract

This paper reviews the research progress on ionogels in flexible pressure sensors. Ionogels comprise solid carrier networks and ionic liquids (ILs) dispersed therein and have good non-volatility, high conductivity, thermal stability, a wide electrochemical window, and mechanical properties. These characteristics give ionogels broad application prospects in wearable electronic devices, intelligent robots, and healthcare. The article first introduces the classification of ionogels, including the classification based on ILs and solid carrier networks. Then, the preparation methods and processing technologies of ionogels, such as the direct mixing method, in situ polymerization/gel method, and solvent exchange method, are discussed. Subsequently, the article expounds in detail on the properties and modification methods of ionogels, including toughness, conductivity, hydrophobicity, self-healing, and adhesiveness. Finally, the article focuses on the application of ionogels in flexible pressure sensors and points out the challenges faced in future research. The language of this mini-review is academic but not overly technical, making it accessible to even researchers new to the field and establishing an overall impression of research. We believe this mini-review serves as a solid introductory resource for a niche topic, with large and clear references for further research.

## 1. Introduction

The term ionogel was first coined by Vioux et al., who successfully prepared ionogels in 2005 by confining ILs in an inorganic matrix [1,2]. Ionogels consist of networks of solid carriers and ILs dispersed in them. When the ionogel is subjected to pressure, the ions within the gel migrate in response to the electric field, resulting in the formation of an electric current that conducts electricity. This new sensing mechanism, based on the migration of ions within a material to convert mechanical stimuli such as pressure into electrical signals, is called the piezoionic technique [3,4,5]. The high ionic conductivity of ionogels ensures efficient ion transport, enabling rapid response to external stimuli such as pressure [6,7]. This is critical for applications in flexible pressure sensors, where the ability to detect and transduce subtle changes in pressure into measurable electrical signals is essential [8,9]. Different cations in the ILs can form about 1018 species by binding different anions [6,10,11]. The ILs have bulky side groups that give the ionogels mechanical flexibility, stretchability, and broad temperature stability [12]. The mechanical flexibility and stretchability of ionogels allow them to conform to complex surfaces and maintain stable performance under dynamic conditions, which is particularly important for wearable devices and electronic skin applications [13]. The wide electrochemical window and thermal stability of ionogels further enhance their reliability in extreme environments [14,15]. Additionally, these side chains also confer a variety of excellent properties and unique design abilities to the ionogels. The functionality of ionogels is modulated by selecting solid carrier networks and ILs with different properties [16,17]. The ionogels are made to have excellent properties such as good nonvolatility, high conductivity, thermal stability, wide electrochemical window, and mechanical properties [18]. Based on these excellent properties, ionogels have a relatively wide range of applications in wearable electronic devices, intelligent robots, and healthcare [6,19,20,21,22,23,24]. It is precisely because of the wide variety of ILs and their very different physicochemical properties that ionogels also possess excellent structural tunability [25,26]. Therefore, once reported, ionogels have attracted a great deal of research from many scholars.

**Figure 1:**
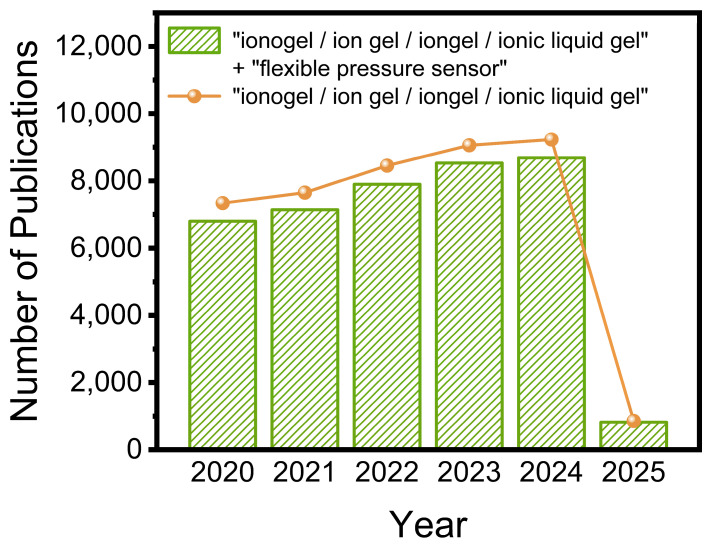
Number of publications dealing with ionogel electrolytes from the year 2020 to March 2025. The keywords used for the search in Scopus are “ionogel”, “ion gel”, “iongel”, “ionic liquid gel”, and “flexible pressure sensor”.

In the past five years, 42,599 research papers on ionogels have been published. Among them, there are even as many as 36,062 papers on flexible pressure sensors, as shown in Figure 1. During our research, we discovered that ionogels have not yet established a unified, fixed nomenclature in the academic community. Researchers have employed multiple synonymous terms. Consequently, we not only searched the Web of Science with “ionogel” and “flexible pressure sensor” as keywords, but also selected several common names as keywords for searching, such as “ion gel” [27], “iongel” [28,29], and “ionic liquid gel” [30]. As can be seen in Figure 1, “flexible pressure sensor” represents one of the largest fields of ionogels research, and this field has experienced explosive growth over the past 5 years. Therefore, it is important to summarize the work of the last 5 years and provide the reader with new insights into future developments.

In order to facilitate the development of more innovative ionogels in our next work, in this mini-review, we summarize the classification of ionogels, preparation methods and processing techniques, properties, and modification methods. In the last part of the review, we focus on the application of ionogels in flexible pressure sensors and mention the challenges to be faced in future research on ionogels. Overall, this review presents recent advances in ionogels and establishes guidelines for the design of ionogels for flexible pressure sensors.

## 2. Classifications of Ionogels

Ionogels can be classified in a variety of ways and can generally be categorized based on the ILs and the solid carrier networks [26].

### 2.1. Types of Ionic Liquid

**Figure 2:**
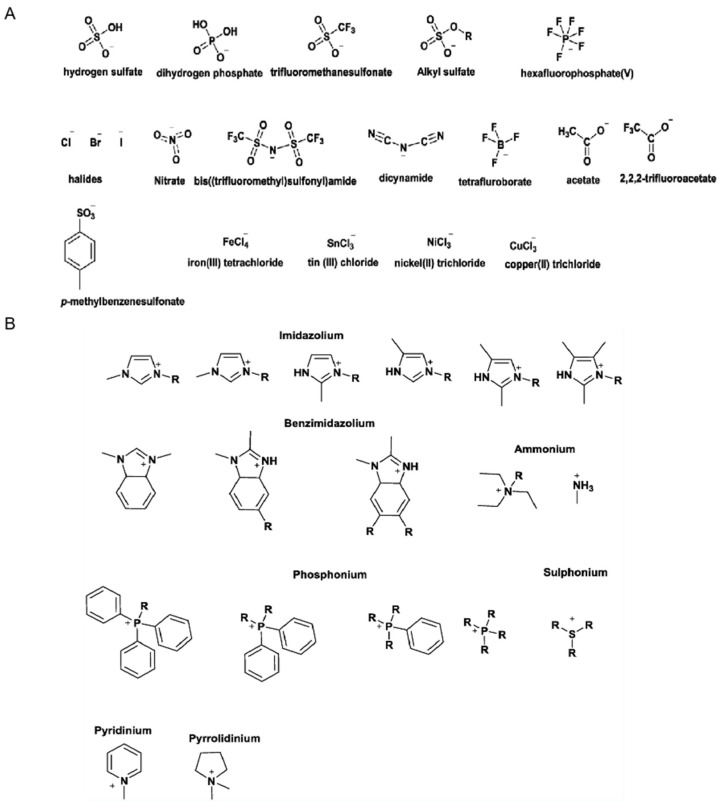
(**A**) Anions and (**B**) cations used commonly in IL synthesis [32].

ILs are generally composed of organic cations and organic/inorganic anions [31]. Anions and cations commonly used in ILs are shown in Figure 2 [32]. Depending on the role of the ILs, ionogels can be divided into two types. One is the solvent type, where the ILs act as the solvent. The other is the building block type, where ILs serve as building blocks, and ionogels containing this type of IL are commonly referred to as polyionic gels [33]. When the ILs act as the solvent, the cations and anions of the ILs can move freely to some extent within the constructed ionogels. This type of ionogel retains the main characteristics of the ILs and is easy to process. Huang et al. used a choline chloride/ethylene glycol (ChCl/EG) deep eutectic solvent (DES) to prepare DES-ionogel@SiO_2_ stationary phase material, synthesizing the ionogels in a green DES medium and modified silica gels. When ILs serve as building blocks, they participate in the construction of ionogel network systems, leading to polyionic gels with superior mechanical properties [34]. In a related study, Zhou et al. utilized low-molecular-weight gelling agents to directly solidify ILs, producing supramolecular ionogels with excellent mechanical and electrical properties [35]. However, compared to solution-type ILs, the building block-type ions cannot move freely, resulting in relatively weaker conductivity [36].

### 2.2. Types of Network Structures

**Figure 3:**
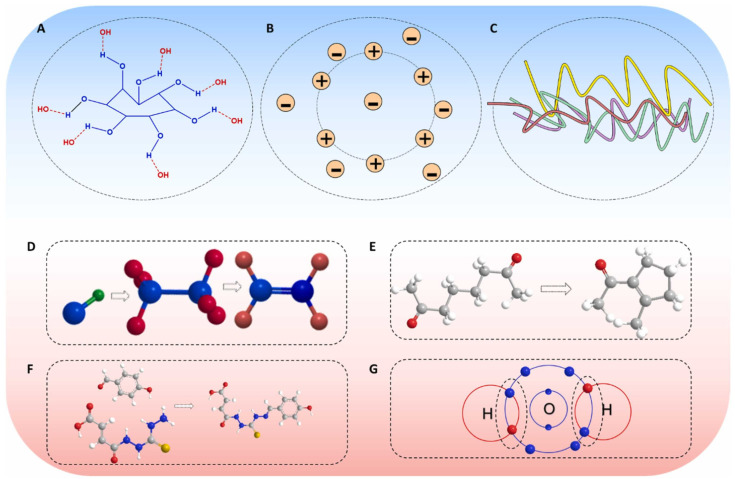
Schematic representation of physical cross-linking modes such as (**A**) hydrogen bonding, (**B**) ionic interactions, and (**C**) chain entanglement. Schematic representation of chemical cross-linking modes such as (**D**) free radical polymerization, (**E**) condensation reactions, (**F**) Schiff base reactions, and (**G**) dynamic covalent bonding [42].

Substances that can serve as the solid carrier networks for ionogels include organic polymers [37], organic small molecules [38,39], oxides [40], and carbon nanotubes [41]. The network structures of ionogels are diverse, and currently, based on the differences in the forces used to form the cross-linked networks, ionogels can be classified into two major categories: physical cross-linking (Figure 3A–C) and chemical cross-linking (Figure 3D–G) [42]. Physically cross-linked networks refer to cross-linked structures formed by physical forces such as hydrogen bonds [43,44], ionic interactions [45], and chain entanglements [46]. These forces are generally weak, making the formation and dissociation of the cross-linked structures quite easy; thus, physical cross-linking is usually reversible [47,48,49]. Common methods for chemically cross-linking ionogels include radical polymerization cross-linking [50], condensation cross-linking [51], Schiff base reactions [52], and dynamic covalent bond cross-linking [53]. Chemical cross-linking, compared to physical cross-linking, possesses stronger forces and binding strength; thus, the cross-linked structures are often more resistant to disruption and irreversible [42]. Physical cross-linking and chemical cross-linking each have distinct characteristics, with their advantages and also significant limitations. For instance, gels prepared solely through physical cross-linking generally exhibit lower mechanical strength and poorer gel formability, whereas in gels formed solely through chemical cross-linking, although they have higher mechanical strength and structural stability, the high density of covalent cross-links reduces the flexibility and mobility of the chain segments, leading to increased brittleness of the gels.

### 2.3. Types of Design Strategy

As the application value of ionogels becomes increasingly prominent and their application fields continue to expand, single-cross-linked ionogels can no longer meet the daily usage demands of force-bearing components. Therefore, how to design the cross-linked structure of gel networks and further enhance the mechanical properties, electrical conductivity, and durability of gels has become a research hotspot in the field of ionogels. To address these issues, a large number of researchers have proposed composite cross-linked networks as a method for improvement and resolution. Common composite cross-linked network structures include double-network structures [54], sliding-ring structures [55], and nanocomposite structures [56].

#### 2.3.1. Double-Network Gels

**Figure 4:**
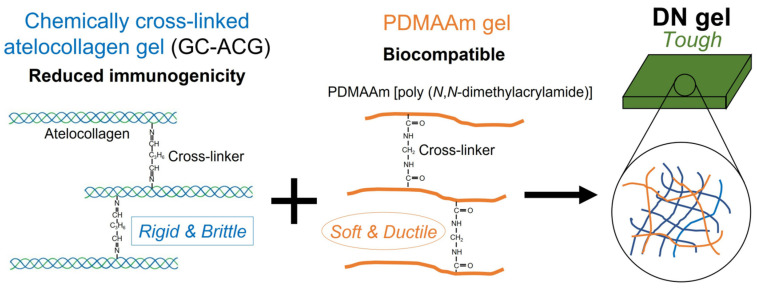
Schematic of the double-network gel structure [60].

The concept of double-network gels was first proposed by Gong et al. Double-network gels are composed of two polymer networks with different cross-linking forms and cross-linking strengths [57]. Compared with gels with single cross-linking forms, double-network gels take into account both mechanical strength and mechanical toughness, and broaden the design ideas of high-performance ionogel materials [58,59]. As shown in Figure 4, the internal networks of double-network gels are composed of highly cross-linked short-chain rigid networks, generally covalently cross-linked, and loosely cross-linked curled flexible networks, generally physically cross-linked [60,61]. When subjected to external stretching and deformation, these two different networks can perform their respective functions and cooperate to resist the destruction of the gels by the external environment, thereby achieving high mechanical properties of the gels. The rigid first network can undergo rapid fracture at the initial stage of stretching. It acts as a “sacrificial bond” to consume a large amount of external energy, thereby protecting the gel’s skeleton from being damaged by external forces. During the stretching process, the polymer chains in the flexible second network gradually change from being curled to straight chains. At the same time, the cross-linking points are constantly dissociating and generating, thereby continuously dissipating energy. Overall, the network design and energy dissipation mechanism of the double-network gels is to use the first network to enhance Young’s modulus and strength of the gels as a whole, and the second network to improve the toughness and ductility of the gels. Excellent mechanical properties make the application value of double-network gels increasingly prominent. With the in-depth research on double-network gels in recent years, ionic conductive double-network gels have also been successfully developed and widely used in fields such as energy storage, flexible electronics, and soft robots [62,63]. In a relevant study, Hao et al. introduced polyethylene terephthalate (PET) and polyurethane into 1-ethyl-3-methylimidazolium dicyanamide salt ([EMI][DCA]) to prepare PET-based polyurethane double-network ionogels. The ionogels have a tensile strength of 4.2 MPa and a fracture elongation rate of 990%, and the conductivity can reach 0.09 S/cm [64].

#### 2.3.2. Slide-Ring Gels

**Figure 5:**
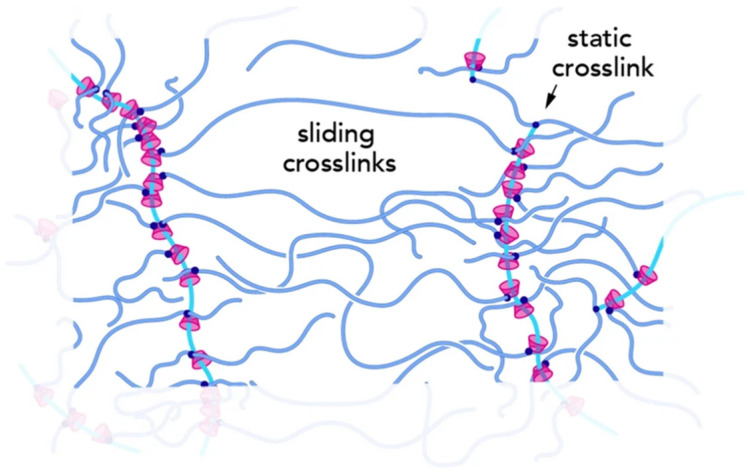
Schematic of the slide-ring gel structure [67].

The sliding-ring gels were originally derived from a kind of composite hydrogel developed by Harada et al., which was based on polyethylene glycol (PEG) and -cyclodextrin (-CD). The researchers first inserted linear PEG into circular -CD to construct hydrogels with a chain-ring structure, laying a foundation for the development of sliding-ring structure gel materials [65]. Subsequently, Okumura et al. further developed the sliding-ring gels. They used PEG chains as guests to penetrate -CD and used high steric hindrance groups to cap both ends of PEG. So that the circular -CD could slide freely on the PEG chain like a pulley. Finally, -CD was covalently cross-linked to construct sliding-ring gels with a unique cross-linking point structure [66]. Different from ordinary covalent cross-linked gel materials, the “8”-shaped sliding-ring cross-linking structure not only endows the sliding-ring gels with stable cross-linking points. But also allows the polymer ring to move freely along the PEG long chain. As a result, the cross-linking points have a certain degree of mobility at the same time, as shown in Figure 5 [67]. This unique structure of the sliding-ring gels gives them a good ability to resist external damage. When subjected to external forces, the cross-linking ring can quickly and evenly distribute the stress to the entire gel network through free movement, thus avoiding local stress concentration and achieving high mechanical performance [68].

#### 2.3.3. Nanocomposite Gels

Nanocomposite gels are a gel material formed by combining nanoparticles as functional cross-linkers with polymer systems through various action mechanisms [69]. Generally speaking, the filled nanoparticles often have a high specific surface area and high strength, and contain some functional groups that can act as cross-linking points. For example, montmorillonite, silica, and graphene oxide. These three materials are the most commonly used nanomaterials for preparing nanocomposite gels at present [70,71]. The mechanical properties of nanocomposite gels are far superior to those of ordinary gel materials. This is mainly because the nanoparticles themselves have high rigidity, and their unique cross-linking methods can effectively increase the cross-linking density of the networks, thereby significantly increasing the modulus and strength of the gel materials. In addition, as cross-linking centers of polymer networks, nanoparticles will cause the bound polymer chains to dissociate and recombine with new surrounding nanoparticles when subjected to external forces. Therefore, during the deformation and stretching process, the cross-linking points of the gel networks are constantly changing and continuously consuming energy, thereby achieving the ultra-high ductility of nanocomposite gels [72]. Due to the simple preparation process and excellent mechanical properties of the prepared gels, nanocomposite technology has been successfully extended to the development of ionogels [73]. Yang et al. developed the crown ether (CE) modified ionic polyvinyl alcohol (PVA) hydrogels. The author introduced CE into PVA (PVA-CE) hydrogels as an ion-selective mobility difference amplifier, which significantly enhanced the pressure-induced voltage response of the hydrogels, achieving a 30-fold improvement in piezoelectric ion coefficient, an ultra-low detection limit of 0.2 Pa and a fast response time of 18.1 ms, providing new possibilities for applications in wearable electronic devices and healthcare fields [74].

## 3. Preparations of Ionogels

Three commonly used ionogel preparation methods are the direct mixing method, in situ polymerization/gel method, and solvent exchange method. Different preparation methods can be realized by choosing different processing techniques according to the structure and end use of the gels. Examples include electrostatic spinning, scratch coating, casting, inkjet printing, 3D printing, etc. [26,42].

### 3.1. Direct Mixing Method

The direct mixing method is the simplest method for preparing ionogels. After forming cross-linked networks, the ILs are restricted by expanding the cross-linked networks to obtain ionogels. Wang et al. fully dissolved lipoic acid (TA) in ethanol and then mixed it with 1-ethyl-3-methylimidazolium ethyl sulfate to prepare super-stretchable ionogels with widely adjustable mechanical and conductive properties, self-healing properties, and tissue-like strain adaptability [75]. Xu et al. directly mixed cellulose with ionogels and polymer skeleton to prepare the IL/poly(N, N-dimethyl acrylamide-methacrylic acid-acrylamide)/cellulose ionogels ternary system. The ionogels exhibited excellent 47.18 kPa adhesion, self-healing capabilities, electrical conductivity, and mechanical properties, which can withstand a stress of 0.48 MPa and a tensile force of 899.69% [76].

### 3.2. In Situ Polymerization/Gel Method

The in situ polymerization/gel method is a commonly used method for preparing ionogels [77,78]. Usually, thermal or ultraviolet light is used to initiate radical polymerization and ring-opening polymerization. Most of the articles reporting the preparation of ionogels by the “one-pot method” reported recently have used this method. Yu et al. mixed the first cross-linking network, the second cross-linking network, and the IL evenly together and prepared a double-network ionogel film by heating and ultraviolet irradiation. This film has a tensile strength of 1.5 MPa and a breaking elongation rate of 120% [79]. Wang fully mixed ethylene glycol diacrylate, polymer monomers, 2-hydroxy-2-methylpropiophenone, 1-ethyl-3-methylimidazolium bis(trifluoromethyl sulfonyl)imide ([EMIM][TFSI]), and hexafluoro isopropanol evenly, and carried out patterned in situ copolymerization reaction under 365 nm ultraviolet irradiation to prepare a flexible film sensor for detecting dimethyl methyl phosphonate [80].

### 3.3. Solvent Exchange Method

Some monomers that are insoluble in ILs can be prepared into ionogels through solvent exchange [81,82]. The solvent exchange method usually prepares polymer cross-linked networks firstly using water or organic solvents. Then, it is soaked in ILs to form ionogels through solvent exchange. Kim et al. formed cross-linked networks of PVA in a mixed solvent of dimethylformamide and dimethyl sulfoxide (DMSO) with a volume ratio of 6:4. Then, through the solvent exchange method, [EMI][DCA] replaced the organic solvent, and ionogels were prepared using a typical solvent exchange method. These ionogels have a tensile stress of 100 MJ/m^3^ and a tensile strain of >1000%, and the conductivity can reach 20.5 mS/cm [83]. Ren et al. soaked the gels containing methanol as the first solvent in 1-propyl-3-methylimidazolium tetrafluoroborate at 80 °C under vacuum for solvent exchange and gradually removed methanol to prepare a super-tough click ionogel [84].

## 4. Properties and Modification Methods of Ionogels

### 4.1. Toughness

In certain application environments, it is essential for ionogels to simultaneously exhibit substantial strength and excellent toughness. Commonly employed methods for enhancing strength and toughness include the design of dynamic cross-linked networks [85], the construction of double or multi-network structures [7], nano-composite reinforcement [8], optimization of solvent and monomer cross-linking strategies [9], and the establishment of physical cross-links and dissipation networks [13], along with structural bionics and hierarchical design [86]. Li et al. utilized halide metal salt ILs, such as 1-butyl-3-methylimidazolium bromide and anhydrous metal chloride, to create double cross-linked networks with PVA through dynamic hydrogen bonds and metal coordination bonds. The modified ionogels achieved a fracture stress of up to 63.1 MPa, a strain of 5248%, and toughness of 1947 MJ/m^3^, significantly surpassing that of conventional metal materials [87]. Wang et al. developed a phase separation structure in IL via the copolymerization of acrylamide (AM) and acrylic acid (AA), resulting in a fracture strength of 12.6 MPa and a toughness of 24 kJ/m^2^, along with self-healing properties [7]. Zhan et al. employed choline chloride-based DES and integrated them with the hydrogen bond networks of PVA to produce ionogels with a strength of 31.53 MPa and a toughness of 203.38 MJ/m^3^, which also exhibits self-healing capabilities and high ionic conductivity [9]. Inspired by the structure of leaves, Peng et al. constructed micro/nano entanglement networks and combined them with the IL replacement strategy to achieve uniform stress dispersion. The gel modulus was measured at 30.4 MPa, demonstrating no attenuation after 4000 fatigue cycles [86].

### 4.2. Conductivity

The core of enhancing the conductivity of ionogels lies in the optimization of ion transport channels and the interactions at material interfaces. Common strategies for improving the conductive performance of ionogels include the introduction of reversible chemical bonds or microphase separation structures [88], the incorporation of conductive nanomaterials [14], the regulation of ILs and solvent systems [15], the design of interpenetrating networks and microstructures [89], and the introduction of functional monomers or dynamic bonds [15]. Si et al. electrochemically grow tin dendrites within polymer hydrogels to establish interconnected conductive networks, thereby enhancing both conductivity and toughness. Following this modification, the conductivity of the hydrogels exhibits a significant increase [8]. Ji et al. designed urea-based ILs and copolymerized them with AM, yielding ionogels with a conductivity of 1.216 S/m, which exhibits both antibacterial and self-healing properties, making them suitable for biosensing applications [15]. Zhang et al. induced microphase separation through the addition of lithium salt, resulting in the formation of rigid nanodomains and continuous liquid-rich phases. This approach increased the ionic conductivity to 2.18 mS/cm while simultaneously improving the mechanical strength by 718% [90].

### 4.3. Hydrophobicity

Compared to conductive hydrogels, ionogels exhibit fewer hydrophilic groups and possess a degree of hydrophobicity. Nevertheless, the application of ionogels in underwater environments presents significant challenges [91,92]. Common hydrophobic modification techniques include the hydrophobic modification method [93,94], solvent exchange strategy [95], and a high cross-linking density approach [96,97]. By introducing hydrophobic functional groups or constructing densely cross-linked networks, these methods enhance the polar-dipole interactions within the ionogels while minimizing water molecule penetration, thereby achieving hydrophobicity [98,99]. Zhang et al. utilized tetraethyl orthosilicate as the silicon source and trimethylchlorosilane (TMCS) as the modifier. Following the formation of the wet gels, the surface was directly modified using a TMCS/n-hexane solution, resulting in the preparation of hydrophobic SiO_2_ gels through atmospheric drying. The measured contact angle was 142°, indicating excellent hydrophobicity [100]. Wei et al. developed novel ionogels with a high cross-linking density using a completely hydrophobic structure, which is employed in wearable underwater sensors and communicators, demonstrating long-term underwater stability [101].

### 4.4. Self-Healing

At present, the development of self-healing gels is generally based on the mobile phase theory. It requires that the prepared gels have dynamically reversible cross-linked networks and a freely movable mobile phase. In short, when the gels are damaged by the outside world, the polymer chains in the damaged area can move and form new cross-linking points with other polymer chains through reversible interactions, thereby realizing the repair of the damaged area [102]. Self-healing materials are generally divided into two categories: external self-healing and internal self-healing [103]. The external self-healing process depends on the introduction of pre-embedded repair reagents, which are usually carried in the form of microcapsules or microvascular fibers and release the repair reagents when broken and perform bonding reactions on the damaged surface [104]. The internal self-healing process depends on reversible interactions formed by non-covalent bonds, such as hydrophobic association, hydrogen bonds, host–guest interactions, or reversible covalent bonds, such as disulfide bonds, boronate ester bonds, and acyl hydrazone bonds, between polymer chains [105,106]. Yan et al. prepared ionogels with a dynamic cross-linked structure by using a strategy combining physical interactions and entangled networks. The polymer-entangled microspheres in these ionogels are deformable physical cross-linking points, which can not only effectively improve the mechanical stability and mechanical elasticity of the ionogels but also reduce stress concentration and improve the fatigue resistance of the gels. In addition, the electrostatic interaction between polymer microspheres and ILs, and the reversible hydrogen bonds between ILs and polymer networks, can endow the ionogels with self-healing properties. Although non-covalent interactions are usually more reversible, they often have weak binding forces and affect the overall mechanical strength of the gels. Therefore, researchers have developed self-healing ionogels based on covalent interactions [107]. Sook et al. reported the synthesis of multi-responsive, self-healing, and air-stable ionogels. The ionogels contain disulfide bonds. Under the heating or irradiation of near-infrared lasers, the self-healing of the ionogels is triggered through the reorganization of hydrogen bonds and the interaction of thiosulfates [108].

### 4.5. Adhesion

The core of improving the adhesion performance of ionogels lies in the optimization of interfacial interaction and the design of dynamic networks [109]. In addition, hydrophobicity, cohesion strength, and environmental adaptability also need to be taken into account. Common methods include introducing supramolecular interactions [110], constructing bicontinuous phase structures [111], introducing functional groups [12], and introducing nanocellulose fillers [112]. Xiong et al. achieved strong adhesion by designing ionogels with supramolecular interactions and adjusting the cohesive energy and interfacial energy of the ionogels. The adhesion strength of the modified ionogels to glass reached 24.4 MPa [109]. Wang et al. prepared ionogels with high transparency and strong underwater adhesion performance by constructing bicontinuous phase structures. The ionogels exhibited excellent adhesion performance in the underwater environment and could firmly adhere to the surfaces of various substrates [113]. Zheng et al. designed programmable adhesive ionogels by introducing catechol groups. This ionogels significantly changes its adhesive property under the preset temperature trigger, and the adhesive force is 600 N/m [12]. Zhao et al. introduced graphene into photocuring 3D-printed ionic hydrogels to enhance the interfacial interaction through the high specific surface area of graphene. The prepared ionogels have an adhesive strength of 121 kPa in a dry environment [112].

### 4.6. Freezing Resistance

The glass transition temperature of ILs in ionogels is generally low. Therefore, their antifreeze performance is better than that of other conductive gels [114]. With the rapid development of science and technology, higher requirements are put forward for the low-temperature resistance performance of ionogels. It is even necessary to work in extremely cold environments [115]. The core of improving the antifreeze performance of ionogels lies in reducing the freezing point, inhibiting the formation of ice crystals, and enhancing mechanical stability at low temperatures [116]. The commonly used strategies to improve the antifreeze performance of ionogels are to introduce solvents or additives with low solidification points [117], construct dynamic and reversible chemical bonds [118], imitate biological antifreeze mechanisms [119], and add nano-materials to improve the thermal conductivity of the gels [117]. Li et al. were inspired by the antifreeze proteins of Antarctic fish and introduced zwitterionic groups, such as sulfonic acid groups and quaternary ammonium salts, into the ionogels. By electrostatic interaction, the ordered arrangement of water molecules is disrupted, and the formation of ice crystals is inhibited. The ionogels have no frost adhesion for a long time at −10 °C and still maintain antifreeze properties even at −49 °C. It is suitable for polar or aerospace fields [119]. Ye et al. compounded cellulose nanofibers (CNFs) with DMSO and prepared PVA-CNF organic hydrogels. The conductivity of the hydrogels still reaches 1.1 S/m at −70 °C and is suitable for flexible circuits in polar environments [14].

## 5. Applications of Ionogels as Flexible Pressure Sensors

Ionogels have wide applications as flexible pressure sensors, covering multiple fields such as wearable electronic devices [120], intelligent robots [121], and healthcare [122].

### 5.1. Classification and Key Parameters of Flexible Pressure Sensors

**Figure 6:**
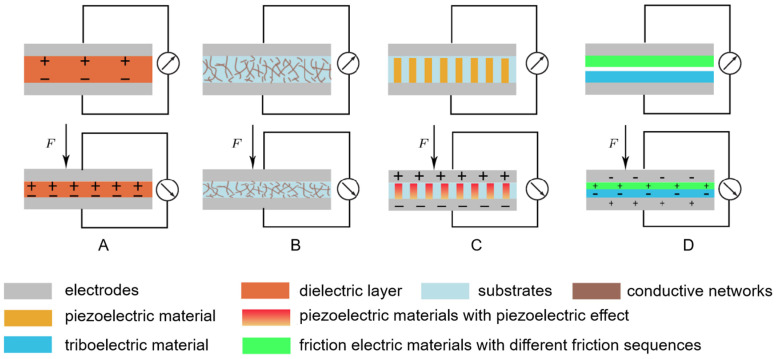
Different kinds of pressure sensors: (**A**) Capacitive pressure sensor; (**B**) Resistive pressure sensor; (**C**) Piezoelectric pressure sensor; (**D**) Triboelectric pressure sensor [123].

Flexible pressure sensors are mainly divided into four types according to the sensing principle, namely, capacitive, resistive, piezoelectric, and triboelectric. They are usually composed of two layers of flexible electrodes and an intermediate functional soft material. The schematic diagram is shown in Figure 6 [123].

Capacitive pressure sensors generally consist of electrodes and dielectric layers. It belongs to a parallel plate capacitor, and its capacitance value is $C=\varepsilon_{0}\varepsilon_{r}A/d$. Among them, $\varepsilon_{0}$ is the vacuum dielectric constant, $\varepsilon_{r}$ is the relative dielectric constant, A is the effective area of the electrode, and d is the spacing between the plates. For devices composed of soft materials, these three variables are all easily affected by pressure, and the magnitude of pressure can be measured according to the change in the capacitance signal. Capacitive pressure sensors can detect static forces. Ordinary flexible capacitive pressure sensors have the advantages of low energy consumption, small signal drift, and high response repeatability [124]. 

Resistance pressure sensors convert pressure changes into changes in resistance or current. According to the definition of resistance R=ρL/S. Among them, ρ is the resistivity, L is the length, and S is the cross-sectional area. Its sensing mechanism is simple, the structure and preparation process are simple, and the energy consumption is small. Therefore, it has attracted extensive attention from researchers [123].

The working principle of the piezoelectric pressure sensor is to convert pressure signals into voltage signals. Its sensing mechanism originates from the piezoelectric effect of piezoelectric materials. The most important parameter used to describe the piezoelectric properties of piezoelectric materials is the piezoelectric constant. This parameter reflects the ability of piezoelectric materials to convert the received mechanical energy into electrical energy. Generally, the larger the parameter is, the better the piezoelectric performance of the piezoelectric pressure sensor is. Since the piezoelectric pressure sensor can generate an internal voltage when subjected to external pressure, the magnitude of the generated voltage is related to the strain. Therefore, this kind of sensor has great potential in the field of passive pressure sensors. The piezoelectric pressure sensor has excellent performance in measuring dynamic pressure, and the response time can reach the microsecond level. However, there are still some difficulties in measuring static pressure [125]. The commonly used piezoelectric materials mainly include polymers and inorganic materials. For example, lead zirconate titanate [126], ZnO [127], and polyvinylidene fluoride (PVDF) [128].

The triboelectric pressure sensor appears later than the first three sensors. Unlike the resistive and capacitive sensors, it does not need power. Unlike the piezoelectric sensor, it has a wider range of optional materials. The triboelectric pressure sensor utilizes the principle of triboelectricity and has the advantages of low cost, simple preparation process, and high output voltage.

### 5.2. Applications in the Field of Wearable Electronic Devices

The high sensitivity, high light transmittance, strong adhesion, and good biocompatibility characteristics of ionogels enable the device to have strong plasticity, closely fit the human body, and accurately monitor subtle movements such as facial expressions and large joint bending [129,130]. Its excellent conductivity and good mechanical properties enable the device to maintain stable performance during repeated stretching and compression [131]. In addition, the precursor solution of ionogels has low viscosity and fast photocuring speed, which can be well adapted to the photocuring 3D-printing system to achieve high-precision printing [130]. Most ionogels possess good transparency, which can meet users’ higher visual requirements. Zeng et al. used 3D-printing technology to prepare highly transparent ionogels that can monitor and distinguish various subtle movements of the human body including facial expressions, limbs, and joints [129]. Sun et al. developed ionogels with high transparency, self-healing ability, and a tensile strain of 300% by using a dual physical cross-linking method of hydrogen bonds and dipole–dipole interactions. The sensitivity is 3.06 kPa^−1^ and it is suitable for wearable energy harvesting and physiological signal monitoring [132].

### 5.3. Applications in the Field of Intelligent Robots

The excellent properties such as multimodal sensing and the self-healing ability of ionogels make them have important application value in the field of electronic skin [133]. By introducing dynamic covalent bonds or non-covalent bonds, ionogels can self-heal after being damaged and restore their conductivity and mechanical properties [134]. Jung et al. proposed an ion-based self-healing electronic skin with very high fracture strength and toughness, creating a comprehensive self-healing electronic skin suitable for soft robot applications [133]. Similarly, Xia et al. developed a multimodal, ultrasensitive, biomimetic electronic skin based on gradient micro-tapered ionogels. It can utilize both the ionic capacitive effect and the triboelectric effect simultaneously, achieving sensing performance with a high sensitivity of 357.56 kPa^−1^, a low detection limit of 0.47 Pa, and a wide linear detection range of 0–500 kPa. This electronic skin can accurately monitor the movement of human finger joints and convert it into letter information on a virtual keyboard, demonstrating the great potential of ionogels in human–computer interaction in the field of intelligent robots and artificial intelligence [135]. The rapid response time and high sensitivity of ionogel-based flexible pressure sensors enable intelligent robots to capture dynamic signals in real time, which is crucial for real-time feedback and precise control of intelligent robots in dynamic environments. The self-healing properties of structure and function not only reduce the manufacturing costs of intelligent robots but also propel robotics technology towards sustainable development.

### 5.4. Applications in the Field of Healthcare

The tunable framework structure of ionogels enables effective drug loading while protecting drug activity and enhancing drug stability. Ionogel-based flexible pressure sensors also possess sensitive tactile perception and adhesion capabilities, allowing for precise localization of drug release sites. These advantages, combined with microcurrent control, facilitate multifunctional and precise controlled drug release [136]. In addition, ionogels are also used to enhance the dissolution of poorly soluble drugs, thereby improving drug absorption and bioavailability. For example, Shukla et al. dissolved acyclovir in a hydrophilic IL containing strong hydrogen bond acceptors to prepare drug-loaded ionogels. This method significantly improved the dissolution of acyclovir (>10%) [137]. Bhamble et al. developed a type of ionogels based on choline geranate (CAGE), which can effectively improve the solubility and transdermal bioavailability of Etodolac (ETO). The solubility of ETO in the ILs of CAGE is about 19,600 times higher than that in water. The ionogels exhibit the characteristic of continuous release for up to 48 h and show about 1.3 times higher flux and about 1.5 times higher permeability coefficient in vitro permeation studies than the hydrogels containing ETO [138]. Ionogels with good adhesion and biocompatibility can enhance the transdermal permeation ability of drugs, achieve local or systemic therapeutic effects, and reduce toxic and side effects [139]. Muzammil et al. developed ionogels based on 3-methyl-1-(hexadecyloxycarbonylmethyl) imidazolium bromide and used them to load doxorubicin. Under the conditions of 37 °C and pH = 5.0, about 82.3% of doxorubicin was cumulatively released within 50 h [140]. The excellent biocompatibility of ionogels can significantly reduce patients’ rejection reactions. Although there have been no reports on the release of drugs from ionogels in vivo, the good biocompatibility of ionogels suggests their potential for long-term use in the body, which could greatly reduce patient rejection reactions. While there is currently no reported research on the release of drugs from ionogels in vivo, with further research and technological advancements, ionogels are expected to play a more significant role in the field of in vivo drug delivery [137,138,139].

The main parameters related to some typical ionogel-based flexible pressure sensors are presented in Table 1 for reference. In summary, ionogel properties are governed by synergistic effects between ILs and solid carriers. When fabricating flexible pressure sensors, researchers often choose solid carrier networks like thermoplastic polyurethane (TPU), PVA, PU, poly (vinylidene fluoride-co-hexafluoropropylene) (PVDF-HFP), acrylamide (AAm), N, N-dimethyl acrylamide (DMAA) and ILs like 1-n-Butyl-1- methylpyrrolidinium bis (trifluoromethyl sulfonyl) imide (BMP-NTf2), CE, [EMIM][TFSI], 1-ethyl-3-methylimidazolium trifluoro methane sulfonate ([EMIm]OTf). The sensors listed in Table 1 pursue different characteristic properties, adopt different overall structures, and employ different preparation methods, leading to significant parameter variations. It can also be observed that ionogels, when used as flexible pressure sensors, exhibit an antagonistic relationship between high sensitivity, detection limit, response time, and response range. Researchers need to make selections of materials and preparation methods based on their requirements.

## 6. Challenges of Ionogels as Flexible Pressure Sensors

Although ionogels have gained much attention and a variety of applications in the past decades as flexible pressure sensors, some challenges related to ionogels still exist and need to be focused on and addressed [143,144].

First, as researchers further explore low-altitude, deep-sea, and outer space environments, new materials are required to perform under extreme conditions [145]. For instance, high-speed flight subjects materials to airflow impact, deep-sea environments impose high hydrostatic pressure and saltwater corrosion, and outer space demands resistance to vacuum and extreme temperature variations [7,146]. Those necessitate ionogels to possess a certain degree of scalability, allowing for modifications based on application requirements. Such as improvements in adhesion, toughness, long-term stability in water, and corrosion resistance of ionogels are essential. Additionally, it is crucial to enhance their decomposition temperature further and lower their glass transition temperature [147]. It is difficult to maintain the balance of environmental tolerance. Antifreeze and dehydration resistance designs often require the introduction of additives, but this operation may sacrifice the conductivity or mechanical properties of ionogels. For example, although the introduction of a high-concentration CaCl_2_ solution can increase the antifreeze property of ionogels to −49 °C, it will reduce its sensitivity [148].

Second, leakage of ILs and signal drift remain technical bottlenecks. Although dynamic cross-linking networks can suppress leakage, the creep of molecular chains during long-term use may still lead to signal drift. For example, traditional ion-electron type sensors have a drift of 30% within 10 min. Although the new polyelectrolyte material reduces the drift to 1%, it still needs to be further optimize network uniformity [149,150].

Third, the manufacturing process is complex, and the cost is high. Although 3D-printing technology with high precision can achieve complex structures, the equipment cost is high, and the synthesis process of ionogel slurry is complex, which limits large-scale production [148].

Finally, there are many multi-modal signal interferences, and they are difficult to analyze. Sensors integrating multiple functions such as pressure and temperature are easily affected by signal coupling interference. For example, Ma et al. developed a flexible pressure sensor capable of sensing micro-pressure. This sensor relies on the edge electric field effect. Changes in environmental humidity may affect its dielectric constant, and anti-interference algorithms need to be developed [150]. Apart from the aforementioned challenges, there are also issues regarding biocompatibility and long-term implant safety that need to be examined. Although ionogels have been used in medical monitoring, the toxicity and degradation products of their long-term implantation still need to be verified. For example, the biodegradation rate of intravascular sensors needs to be synchronized with tissue repair to avoid the risk of secondary surgery [151,152].

## 7. Conclusions

This mini-review discusses the classification of ionogels from two aspects: ILs and cross-linked networks. It also summarizes the preparation methods and processing techniques, properties, and modification methods of ionogels. The properties of ionogels such as natural conductivity, good electrochemical properties, high thermostability, and anti-freezing capability were also discussed, which endow the ionogels with broad application prospects. Finally, we also discussed the applications of ionogels in wearable electronic devices, smart robots, and healthcare. Although there are still many challenges and dilemmas to be solved for the application of ionogels in flexible pressure sensors, it can be seen through the summary of this paper, mainly from the structural design, cross-linking networks, nanocomposites, etc., that ionogels can be targeted for modification, so that the development of ionogels can break through existing dilemmas, with a wider range of application areas.

## Figures and Tables

**Table 1:** The main parameters related to some typical ionogel-based flexible pressure sensors.

#	Ionic Liquid	Solid Carrier Networks	Response Range (kPa)	Sensitivity (kPa^−1^)	Response, Relaxation Time (ms)	Low Detection Limit (Pa)	Strain (%)	Stress/ Compressive Stress (kPa)	Temperature (℃)	Ref.
1	BMP-NTf_2_	TPU	0.6~12	0.033	346, -	—	2620	4.34	30 to 100	[51]
			12~25	0.008						
2	CE	PVA	0.2	684.4	18.1, -	0~1000	390	1220	25	[74]
3	[EMIM][TFSI]	TPU	0~20	3.744	100, -	—	—	—	25	[130]
			20~800	1.689						
4	[EMIM][TFSI]	PVDF-HFP	0.47	357.56	10, 20	0~500	—	—	25	[135]
5	[EMIM][TFSI]	PVDF-HFP	2.5	12.8	20, 30	0~1000	—	—	25	[141]
6	[EMIM][TFSI]	PVDF-HFP	0.5	12.8	60, 50	0~80	—	—	25	[142]
7	[EMIm]OTf	AAm/DMAA	8	1.2	52, 68	0.008~1000	97	990	−30 to 150	[143]

## Data Availability

No new data were created for this work.
